# The Effect of Altitude on Intraocular Pressure in Vitrectomized Eyes with Sulfur Hexafluoride Tamponade by the Friedenwald Method: Rabbit Animal Model

**DOI:** 10.1155/2016/7326160

**Published:** 2016-11-10

**Authors:** Jans Fromow-Guerra, Adriana Solís-Vivanco, Raul Velez-Montoya, Adriana Perez-Reguera, Hugo Quiroz-Mercado, Armando Meza-de Regil, Gabriela Papa-Oliva, Virgilio Morales-Cantón

**Affiliations:** Retina Department, Asociación para Evitar le Ceguera en México IAP, 04030 México City, Mexico

## Abstract

The aim of this study is to assess the change in intraocular pressure after a road trip, in eyes with different levels of filling with gas tamponade. Five rabbit eyes were subject to pars plana vitrectomy and gas tamponade (filling percentage: 25%, 50%, and 100% of nonexpansile SF_6_, 100% saline solution, and 100% room air). A sixth eye was injected with 0.35 cc of undiluted SF_6_ without vitrectomy. Guided by global positioning system, they were driven to the highest point of the highway connecting Mexico City with Puebla city and back, stopping every 300 m to assess intraocular pressure. The rabbit's scleral rigidity and estimation for human eyes were done by using the Friedenwald nomogram. Maximum altitude was 3209 m (Δ949 m). There were significant differences in intraocular pressure on the rabbit eyes filled with SF_6_ at 100%, 50%, 25%, and 100% room air. Per every 100 m of altitude rise, the intraocular pressure increased by 1.53, 1.0046, 0.971, and 0.97 mmHg, respectively. Using the human Friedenwald rigidity coefficient, the human eye estimate for intraocular pressure change was 2.1, 1.8, 1.4, and 1.1 mmHg per every 100 m of attitude rise. Altitude changes have a significant impact on intraocular pressure. The final effect depends on the percentage of vitreous cavity fill and scleral rigidity.

## 1. Background

Increased intraocular pressure (IOP) during the immediate and intermediate postoperative stage is a commonly found complication in vitrectomized patients, with an incidence ranging between 20 and 35% of all vitreoretinal procedures [1]. The mechanism and risk factors include (but are not limited to) the following: older age, aqueous humor dynamics, which is in turn highly dependent on the preoperative eye condition, concomitant scleral buckling, and use of silicon (overfilling) or gas tamponade (overfilling, dilution errors) [2–5]. The incidence of the latter is higher than in the rest, since there are studies reporting between 26 and 59% incidence during the immediate postoperative stage [6–8].

Clinical manifestations vary and include a wide array of manifestations: from a mildly painful eye to severe vision loss, due to acute central retinal artery occlusion or optic disk ischemia, requiring emergency gas aspiration in order to secure adequate retinal perfusion [6]. Several preventive measures have been proposed in order to avoid this complication, like transsurgical systemic hypotensive drugs, topical postsurgical hypotensive drops, and inferior iridectomy, to name a few [9–13].

Regarding the severe increase in IOP due to gas tamponade expansion, histopathological studies have proven that expansion of intraocular gas produces both reversible and irreversible changes to intraocular tissues, depending on the length of exposure and gas dilution, including rupture of the hematoretinal barrier and irreversible retinal damage [14–16].

The availability of modern communication and transport routes (air and land transportation) has changed patient's potential mobility after surgery and therefore the risk for IOP complications on the postoperative stage [17–20]. Early reports about IOP complications during air travel prompted in-deep studies regarding intraocular gas behavior and the role that atmospheric pressure has over patients traveling during the early postoperative period [18, 21–24]. Although there is no specific intraocular gas volume that can be deemed “safe” for intravitreal injection before air travel, one study has suggested that an intravitreal volume of 0.6 cc may be well tolerated; nevertheless, there are plenty of other factors that should be considered first, before making a recommendation [18, 25]. In addition, there is no enough evidence regarding the effect of atmospheric pressure over patient's IOP during land travels, where the patient is subject to different altitudes on a single ride, but with a more gradual variation than that during air travel [2, 26]. Nowadays, as part of regular practice, retina surgeons often encounter foreign patients (local or international) needing retinal procedures, in where the selection of the best available tamponade will be determined more by the travel needs of the patient, rather than the clinical characteristics of the case [27]. Moreover, a routine question like if it is safe for the patient to travel back home is usually answered with empirical information, based on theoretical approximations made from the chemical properties of the tamponade agents (provided by the manufacturers), and not by real-life data like percentage of filling at the end of the case, real expansion of the bubble, gas dilution, local altitude, distance to home, final altitude of the site where the patient is bound for, altitude changes along the road, and means of transportation (car, bus, light rail, and train), all of which can result in a different increase or decrease of intraocular pressure [2, 26].

The global positioning satellite (GPS) system is a radio-based navigation system composed of 24 satellites and earth stations; an earth receiver uses the satellites as points of references for calculation of the precise positioning of an object, making measurements such as latitude, longitude, altitude, and atmospheric pressure, with minimal error range [28–30]. By being aware of such specific values, the retina surgeon will be able to advise the patient properly regarding the limits of his journey back home, depending on the kind of gas tamponade, speed, and type of means of transportation.

Therefore, the aim of this study was to estimate the hypertensive effect, induced by the change in altitude (determined by GPS), in vitrectomized eyes with different percentage of air-fluid exchange and sulfur hexafluoride (SF_6_) on an animal model, and to use that information to estimate the hypertensive effect in human eyes through the Friedenwald tabular method.

## 2. Materials and Methods

This is a prospective, longitudinal, experimental, comparative, and masked study. The study was reviewed and approved by the Asociación para Evitar la Ceguera Institutional Animal Care and Use Committee (IACUC) and the Local Institutional Review Board and Research Committee. All procedures and handling were performed according to the statement for the use of animals in ophthalmic and visual research guidelines, from the Association of Research of Vision and Ophthalmology (ARVO). All procedures were funded by the Asociación para Evitar la Ceguera Research Grant.

After an adaptation period of three weeks of the animal lab, a group of six white New Zealand rabbits (WNZ), with weights ranging between 3800 and 4300 Kg, were included in the study. In five of the rabbits, one eye was randomly selected for a two-port-pars plana vitrectomy. The surgery was done under general anesthesia (Ketamine/xylazine and isoflurane). A 23-gauge infusion cannula was placed in the superior temporal quadrant and a secondary trocar for the vitrectomy hand piece was inserted in the inferior nasal quadrant, 4.0 mm from the scleral limbus. A 20-diopter lens and indirect ophthalmoscope were used as illumination and visualization system. After vitrectomy, an air-fluid exchange was done in three eyes as follows: The first eye had a 25% exchange, the second had a 50% exchange, and the last had a 100% exchange. Subsequently, a 20 cc syringe with SF_6_ at a nonexpansile dilution (18%) was placed on the infusion cannula and 15 cc of its content was injected, in order to ensure a complete air exchange with the gas. Once this procedure was completed, sclerotomies were closed with 7.0 suture and an IOP <20 mmHg was verified. Topical analgesia was achieved with ketorolac drops (every 6 hours) and tobramycin drops and ointment was used as postsurgical antibiotic therapy. The fourth and fifth vitrectomized eyes had the same procedures done. However, the fourth eye received a 100% air-fluid exchange with no gas after it (100% room air) and the fifth was left with only balanced saline solution (BSS [no air-fluid exchange]) after vitrectomy (control). The sixth eye in the study was not vitrectomized. Instead, a 0.35 cc bubble of undiluted SF_6_ (100%) was injected intravitreally, in order to mimic a pneumatic retinopexy (Table 1).

Twenty-four hours after the surgery and under mild sedation with Ketamine/xylazine, all six study eyes were assessed for vitreous cavity's gas fill percentage and anteroposterior axis with a mode B ultrasound (immersion technique, Aviso S, Quantel Medical, Haggerty Lane, Bozeman, MT, US). In addition, central pachymetry (AccuPach VI pachymeter, Accutome, Phoenixville Pike, Malvern, PA, US) and IOP by two different assessment methods, applanation tonometry (Tono-pen, Reichert Tech, Walden Avenue, Depew, NY, US) and indentation tonometry (Schiötz tonometer), were also assessed. All measurements were done in triplicate.

All animals were then transported by land on a van, with a temperature-controlled cabin, through the highway connecting Mexico City and City of Puebla. The trip started from the first toll point (Autopista Mexico-Puebla 15a, San Marcos Huixtoco, latitude: 19.296238/longitude: −98.870535, altitude: 2248 m above sea level) to the highest reported altitude point on the highway (Autopista Mexico-Puebla, Santa María Huexoculco, latitude: 19.336444/longitude: −98.709696, altitude: 3228 m above sea level) and back to Mexico City. During the trip, the following variables were measured every 300 m of rise on altitude (by GPS): IOP with the aforementioned methods (Tono-pen and Schiötz), room temperature, and altitude, directly from the GPS display. All measurements were done in triplicate.

Statistical analysis was performed using descriptive statistics. The difference in IOP measurements, obtained through both methods, was analyzed with the Wilcoxon signed rank test. The correlation between both methods was established using the Spearmen correlation, and the relevant coefficients of determination were obtained. As we found no statistically differences between both methods in all cut points, all the results will be presented with the applanation method. For each case, a linear regression was performed, and the slopes were compared through their 95% confidence intervals methods [31].

Once the results from each study eye had been obtained, the effect on human intraocular pressure was estimated based on the Friedenwald method. Friedenwald stated that a variation in intraocular volume *V*_2_ − *V*_1_ was correlated to a fixed variation of intraocular pressure, *P*_2_ − *P*_1_. The logarithmic transformation of such curve renders a straight line, whose slope represents scleral rigidity (*E*) [32]. Scleral rigidity is a constant and individual factor for each eye. The mathematical formula for this relations is as follows:

(1) $$\begin{array}{c} E=\frac{\log P_{2}-\log P_{1}}{V_{2}-V_{1}}. \end{array}$$

With the results obtained from the Schiötz tonometer and Friedenwald's tabular method, the ocular rigidity of rabbit eyes was set for each study eye. Subsequently, with the above mathematical ratio, and taking into account the average human scleral rigidity (*E*_*h*_) reported by Friedenwald (0.0215) and other authors, the following variables were calculated for each case: (1) rabbits' scleral rigidity (*E*_*r*_) was determined using Friedenwald's tabular method and the IOP results, obtained by the Schiötz tonometer using the 5.5 and 10 gr weights, in which the mean turned out to be 0.1811667. (2) Knowing *P*_2_ − *P*_1_ obtained from the study eyes (where *P*_1_ is the baseline intraocular pressure at 2260 m above sea level; and *P*_2_ is the intraocular pressure at any higher altitude), the log⁡*P*_2_ − log⁡*P*_1_ was determined for each test point (2410 m, 2740 m, 3093 m, and 3198 m). (3) With each study eye's scleral rigidity (*E*_*r*_) and the log⁡*P*_2_ − log⁡*P*_1_ for each test point, the change in intraocular volume was assessed per case and per tested altitude:

(2) $$\begin{array}{c} V_{2}-V_{1}=\frac{\log P_{2}-\log P_{1}}{E_{r}}. \end{array}$$

(4) Once the intraocular volume change for each study eye was known (*V*_2_ − *V*_1_), and by using the same formula as before, the log⁡*P*_2_ − log⁡*P*_1_ for humans was calculated, replacing *E*_*r*_ with *E*_*h*_. The value of *E*_*h*_ was considered according to several scleral rigidity coefficients for human eyes reported elsewhere: Friedenwald: 0.0215, Pallikaris: 0.0126, Dastiridou mean: 0.0224, and Dastiridou max: 0.0343 [33, 34]. The scleral rigidity coefficient Dastiridou min was not included in this calculation because the value was similar to the one reported by Pallikaris et al. [33].

(3) log⁡P2−log⁡P1=V2−V10.0215Friedenwaldlog⁡P2−log⁡P1=V2−V10.0126Pallikarislog⁡P2−log⁡P1=V2−V10.0224Dastiridou  meanlog⁡P2−log⁡P1=V2−V10.0343Dastiridou  max

(5) After obtaining the human log⁡*P*_2_ − log⁡*P*_1_, this relation was quantified into mmHg, instead of logarithmic values. In order to accomplish this, and observing the rules of logarithmic relations, where log_10_⁡*U*/*V* = log_10_⁡*U* − log_10_⁡*V*, the following formula was calculated:

(4) $$\begin{array}{c} 10^{\log P_{2}-\log P_{1}}\;\left(\text{This value renders the}\;\;\frac{P_{2}}{P_{1}}\text{ ratio}\right). \end{array}$$

(6) Finally, if *P*_1_ is the baseline IOP at 2260 m (14 to 16.39 mmHg in study eyes), *P*_2_ for humans per case and tested altitude was determined with the aforementioned ratio.

## 3. Results

### 3.1. Rabbits

Six eyes from six healthy WNZ rabbits were included in the study. None of the study eyes experienced complications during surgery or during the postoperative stage.  Table 2 summarizes the altitudes at which IOP was measured, the maximum altitude reached, and the measured IOP by indentation and applanation method, per case. As shown in Table 2, there was no statistical significant difference among both IOP measurement methods. Nevertheless, it positively shows high correlation and coefficients of determination. Accordingly, it may be concluded that both methods are equivalent and consistent between them in this model. Therefore, from now on, only the results obtained by the Tono-pen will be presented.

**Figure 1:**
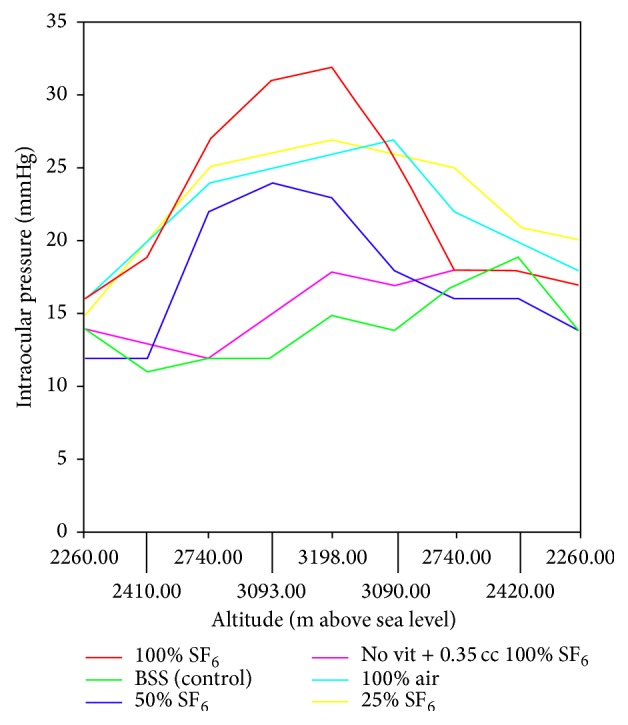
Summary of the changes in intraocular pressure in all study eyes (rabbit). The graph depict changes secondary due to the ascent and descent during the road trip. m: meters. SF_6_: sulfur hexafluoride. BSS: balanced saline solution.

Figure 1 summarizes the IOP variation per case, during the ascent and descent part of the road trip.  Table 2 and Figure 1 show that the IOP variation was similar in the 100% SF_6_, 50% SF_6_, 25% SF_6_, and 100% air study eyes. The rise in altitude was quickly followed by an increase in IOP (although never exceeding 32 mmHg). In addition, the eye with SF_6_ and no vitrectomy and the eye with vitrectomy and BSS did not show any significant IOP increase during the road trip.

In order to establish the impact of altitude variation over the IOP, the correlations between altitude and IOP, as well as the relevant coefficient of determination and linear regressions, were determined on a case-by-case basis.

**Figure 2:**
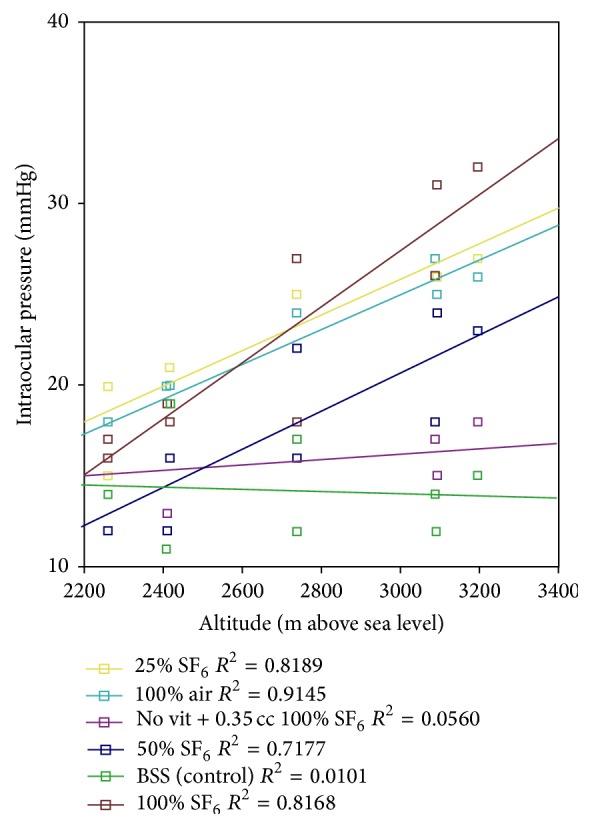
Scatter plot and regression lines from all cases in the rabbit model. m: meters. SF_6_: sulfur hexafluoride. BSS: balanced saline solution.

Figure 2 shows the scatter plot and best fit lines resulting from the regression for each case; the eyes with the steepest slopes (higher impact of altitude over IOP) were (from higher to lesser impact) 100% SF_6_, 100% air, 25% SF_6_, and 50% SF_6_, while the eye with SF_6_ and no vitrectomy and the eye with vitrectomy and BSS displayed a very low slope, thus proving that an altitude variation has no impact on the IOP on the control eye.

**Figure 3:**
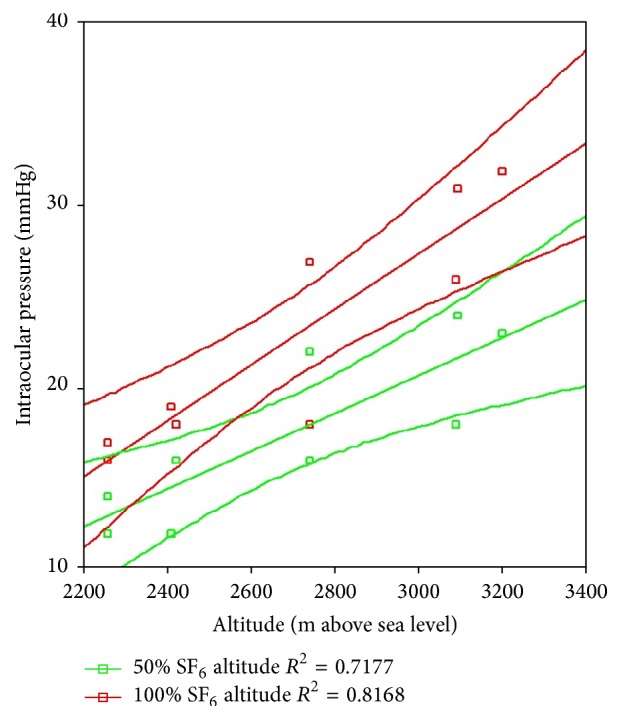
Rabbit model regression lines with 95% confidence interval lines for the study eye with 100 SF_6_ and 50% SF_6_. m: meters. SF_6_: sulfur hexafluoride.

**Figure 4:**
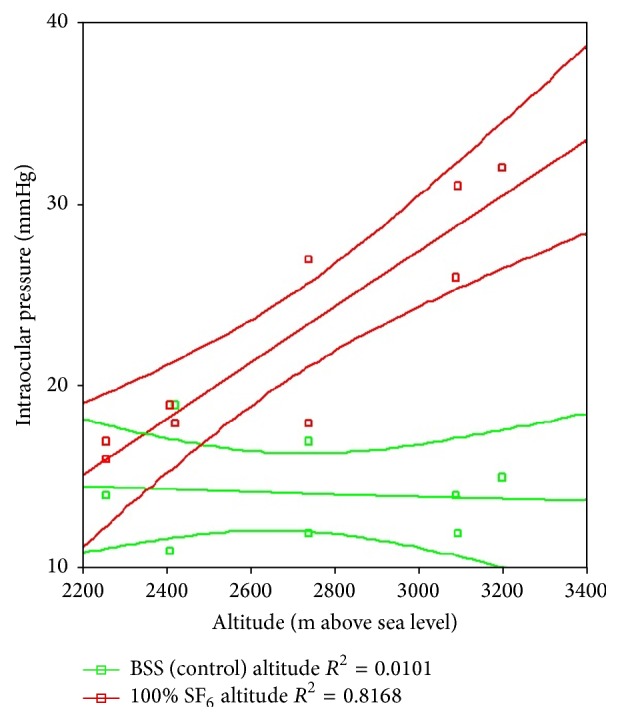
Regression lines with 95% confidence interval lines for the study eye with 100 SF_6_ and BSS. m: meters. SF_6_: sulfur hexafluoride. BSS: balanced saline solution.

Table 3 shows the different regression lines per case, as well as the relevant slopes for each rabbit eye. As noted before, the eye in which IOP increased the most, upon altitude variation, was the eye with vitreous cavity 100% filled with SF_6_: 1.53 mmHg (95% CI: 0.9–2.2) per every 100 m of altitude rise. The eyes with no difference were those with SF_6_ and no vitrectomy and the eye with vitrectomy and BSS. The statistical significance of such differences on each case was set by comparing the cases' confidence intervals, considering 100% SF_6_ case as the benchmark, since it showed the highest increase in IOP. The 100% SF_6_, 50% SF_6_, 25% SF_6_, and 100% air study eye behaved similarly, with nonsignificant differences, while the eyes with SF_6_ and no vitrectomy and the eye with vitrectomy and BSS showed no IOP change in connection with altitude variations. Figures 3 and 4 illustrate these different behaviors in the experimental model for 100% SF_6_ and 50% SF_6_ (similar response) and the controls.

### 3.2. Inference Result for Humans Based on the Friedenwald Method and Other Human Scleral Rigidity Coefficients

Since the aforementioned results pertain to rabbit's eyes, with an ocular rigidity that is different from that of humans, calculations were made in order to determine the approximate IOP changes that could be expected in human eyes, with its own particular ocular rigidity. These inferences are done in order to have an approximation of what can be expected on human eyes and do not represent a precise definitive estimate. As described in Materials and Methods, we established a mean rabbit scleral rigidity (*E*_*r*_) of 0.01811667 (range: 0.0179 to 0.0182); for the human approximations, we used the scleral rigidity coefficients reported by Friedenwald, Pallikaris, Dastiridou mean, and Dastiridou max [33]. The last three were determined using live human eyes. Volume changes were determined by using the experimental formulas in Section 2. The potential changes that might occur in a human eye were determined by replacing *E*_*r*_ with different values for *E*_*h*_.  Table 4 shows the straight lines resulting from the regression used in these estimations for humans, per case and per coefficient and their relevant slopes.

Regarding human estimations and considering just the Friedenwald scleral rigidity coefficient of 0.0215, the cases where IOP increased the most due to altitude variation were those with a vitreous cavity of 100% and 50% filled with SF_6_. The change in the IOP was 2.1 mmHg (95% CI: 1.7–2.5) per every 100 m of altitude rise and 1.8 mmHg (95% CI: 0.6–3.0) per every 100 m of altitude rise, respectively. The eyes with no significant increase in IOP were the eyes with SF_6_ and no vitrectomy and the eye with vitrectomy and BSS (control).

Table 4 also shows the different results of IOP increase according to the different human scleral rigidity coefficients. Despite the variation in the final magnitude of IOP change per 100 m of altitude rise, the general order of the study eyes did not change and the eyes with the higher impact on final IOP were again the eyes with vitreous cavity of 100% and 50% filled with SF_6_. The Dastiridou mean and Friedenwald coefficient displayed a similar behavior, since their total values are similar (0.0224 and 0.0215). The Pallikaris coefficient (0.0126) yielded the lower impact on IOP while the Dastiridou max coefficient yielded the greater impact. For the eyes with vitreous cavity 100% filled with SF6, the latter estimated an IOP increase of 4.4 mmHg per 100 m of altitude rise.

The statistical significance of IOP variation between cases was established again by comparing the cases' confidence intervals, considering 100% SF_6_ case as benchmark since it showed the higher increase in IOP.

**Figure 5:**
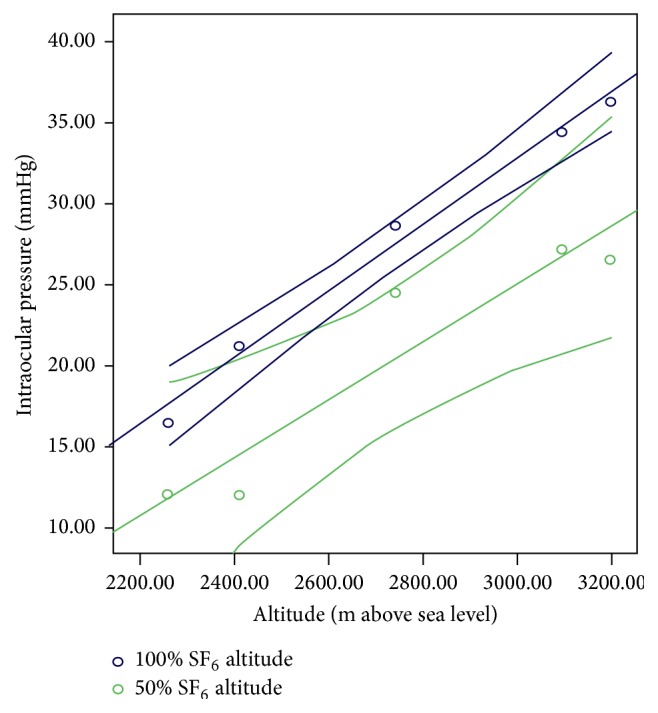
Estimated data for human eyes. Regression lines with 95% confidence interval lines for eyes with 100% SF_6_ and 50% SF_6_. m: meters. SF_6_: sulfur hexafluoride.

**Figure 6:**
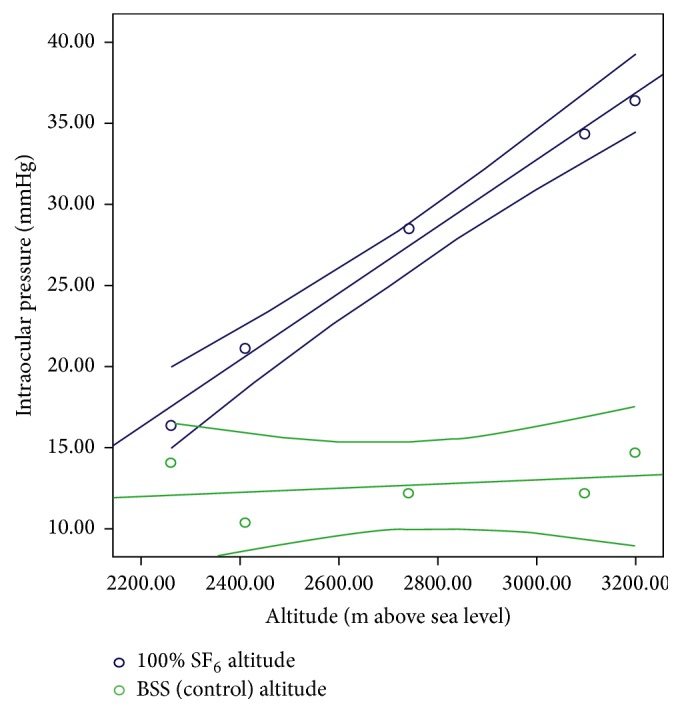
Estimated data for human eyes. Regression lines with 95% confidence interval lines for the study eye with 100 SF_6_ and BSS. m: meters. SF_6_: sulfur hexafluoride. BSS: balanced saline solution.

Estimations for humans revealed that 100% SF_6_, 50% SF_6_, 25% SF_6_, and 100% air eyes showed similar behaviors (nonsignificant differences), while the eye with SF_6_ and no vitrectomy and the eye with vitrectomy and BSS showed no IOP changes in connection with altitude variations (as in rabbits). Figures 5 and 6 illustrate the differences in the estimates for humans between 50 and 100% SF_6_ fill against 100% SF_6_ with no vitrectomy and vitrectomy with BSS.

## 4. Discussion

The use of gas tamponades in vitreoretinal surgery is a common and efficient practice for the treatment of retinal breaks [35]. The half-life of the different compounds (SF_6_, C_3_F_8_, C_2_F_6_, etc.) varies and is subject to different factors [7]. The effect of different intraocular gases on IOP, due to altitude changes while traveling by land, with different vitreous cavity fill percentages has not been comprehensively studied yet. The current experimental model shows the way these variables interact in a comparative animal model, under circumstances that mimic real-life conundrums for patients with vitrectomy and gas tamponade. Along with altitude variations, changes in room and barometric pressure were recorded; however, since the latter two variables are collinear to altitude, we decided to conduct the analysis on altitude solely, as it is easier to measure and understand.

It is common knowledge that, even if used at a proper nonexpandable concentration, an altitude rise and its consequent reduction in atmospheric pressure will induce an expansion effect on gas tamponades which will result in IOP increase [14, 18, 22].

Our results are consistent with previous observations, since IOP increases in all study eyes except for the eye with no vitrectomy and 0.35 cc of 100% SF_6_ and the control eye (BSS). The rise of the IOP had a positive correlation to altitude; however, in contrast to common beliefs and expectations, none of the eyes in the rabbit model exceeded 32 mmHg during the road trip. After calculating the regression slopes for each eye, we realized that the eye who suffered the highest impact on IOP due to altitude variation was the one with the vitreous cavity 100% filled with SF_6_ (+1.53 mmHg per every 100 m of altitude rise). However, this change was not statistically different in relation to the other eyes in the study (50% SF_6_, 25% SF_6_, and 100% air), which also displayed a similar behavior during the road trip. Nevertheless, the trends and differences in slope magnitudes in the rabbit model (change in mmHg per every 100 m of altitude rise; Table 3) showed a directionality toward greater impact on the baseline IOP, depending on the cavity fill percentage and on the type of selected gas tamponade. It is important to highlight that, in this model, the eye filled with 100% room air experienced a significant increase in the IOP, not statistically different from the one observed with the cavity filled with 100% SF_6_. Regarding human eyes approximations, the same behavior was observed with the Friedenwald, Pallikaris, and Dastiridou mean scleral rigidity coefficients. However, with the Dastiridou max coefficient, the IOP increase per 100 m of altitude rise in the eye with the vitreous cavity 100% filled with SF_6_ was more than double the one observed in the eye 100% filled with air. This difference was statistically significant. This result clearly demonstrates the effect that a higher scleral rigidity has over the final IOP and demonstrates that it should be considered in clinical practice when considering patients known to have conditions that increase such variable like in hyperopic patients.

The lack of a significant difference among eyes with different filling percentage may be due to the fact that only one case was done per category (IRB and IACUC restriction for this study). However, these results are able to illustrate the general behavior of intraocular gases and how they are affected by the variables studied herein.

Finally, it is important to consider that, due to the fact that the road trip was done 24 hrs after the surgery, the eye with the undiluted bubble of SF_6_ could still have been going through the expansile phase of the gas. Therefore, the IOP could potentially behave differently on subsequent days.

We are aware that the results from the study depend on different variables; furthermore, they should not be initially and directly extrapolated to human scleral rigidity. We did a mathematical approximation by using different human scleral rigidity coefficients in order to have more useful data for clinicians as well as a broader sense of the most accurate behavior. As a general approach, for an eye with an average scleral rigidity, we suggest using Friedenwald and Dastiridou mean approximations; for eyes with decreased scleral rigidity, the Pallikaris approximation; and for eyes with increased rigidity, Dastiridou max.

Scleral rigidity has been studied before by several authors and it has been determined to be a measure of the ocular wall's resistance to distension [32, 33, 36, 37]. Friedenwald demonstrated in his study that, above 5 mmHg, scleral rigidity is constant and invariable for each eye. He described that scleral rigidity depends on various factors, including the thickness of the eye wall and its radii of curvature [32, 38].

Proof of the relevance of such ratios in everyday practice is the inaccuracy of some devices for IOP measurement, like indentation tonometers in myopic eyes [39, 40]. A lower wall rigidity will yield lower IOP values than the actual ones, making clinicians believe that the eye has normal IOP, when in fact it may have been glaucoma [34]. The opposite occurs in hyperopic eyes, where scleral rigidity is increased [40].

Different methods can be used in order to determine an eye scleral rigidity. In particular, by using Friedenwald's tabular methods, we established that, in our rabbit experimental model, scleral rigidity had a mean of 0.01811667 (range: 0.0179 to 0.0182 [32]). In his original study, Friedenwald determined that the average scleral rigidity of the human eye was 0.0215 [32], a difference of 0.0034 or 4.3% higher than that in our rabbits. It is important to highlight that the greater scleral rigidity in humans is measured on a logarithmic scale; therefore, a difference of 4.3% is significant. After the mathematical inference for humans was done (Table 4), it became clear that, in every case, the impact of the 100 m altitude rise was greater and more significant in humans.

By extrapolating such results, in order to obtain an estimate, using the mean Friedenwald coefficient, a vitrectomized patient at sea level, with a vitreous cavity 100% filled with 18% SF_6_ (nonexpansible dilution), with a baseline IOP of 15 mmHg, who must travel to Mexico City (2260 m above sea level) will behave as follows.

**Example 1 .**

Baseline IOP is 15 mmHg. There is a change of 2.1 mmHg per 100 m of altitude rise. Final altitude is 2260 m above sea level (2260/100 = 22.6). Therefore, 22.6 × 2.1 = 47.46 mmHg of IOP increase. Final IOP will be baseline IOP (15 mmHg) plus 47.46 mmHg = 62.46 mmHg (95% CI: 53.42 to 71.5 mmHg). A final IOP of such magnitude will jeopardize ocular perfusion and probably severe pain.

## 5. Conclusions

In summary, in this experimental animal model, altitude rise showed a significant impact on the IOP of vitrectomized rabbit eyes, with vitreous cavities filled with 100%, 50%, and 25% SF_6_, as well as with 100% room air. There was no significant variation in the IOP of the eye with no vitrectomy and injected with 0.35 cc of 100% SF_6_ (which may mimic what happens in a pneumatic retinopexy) and in the control eye (just BSS). The three most important variables that can impact the IOP while traveling by land are the type of selected gas tamponade, the filling percentage of the vitreous cavity at the beginning of the trip, and scleral rigidity. The estimation for human eyes in this study is approximation, based on this particular model. There are several other covariates to be considered, such as patient's age that might have an impact on scleral rigidity, the axial length, and vasculature. Therefore, caution is advised when extrapolating these results, as further assessment is needed in order to clarify the role of such variables. Each case must be individualized according to each patient's particular characteristics.

## Figures and Tables

**Table 1:** Experimental rabbit model.

Number	Vitrectomy	Gas tamponade	Vitreous cavity fill
1	Yes	18% SF_6_	100%
2	Yes	18% SF_6_	50%
3	Yes	18% SF_6_	25%
4	Yes	100% room air	100%
5	Yes	BSS	100%
6	No	100% SF_6_	0.35 cc (volume)

Summary of the study group and allocation. SF_6_: sulfur hexafluoride. BSS: balanced saline solution.

**Table 2:** IOP at different altitudes and different measurement methods.

Altitude	SF_6_ 100% Tonopen	SF_6_ 100% Schiotz	SF_6_ 50% Tonopen	SF_6_ 50% Schiotz	SF_6_ 25% Tonopen	SF_6_ 25% Schiotz	Air 100% Tonopen	Air 100% Schiotz	Pneum Tonopen	Pneum Schiotz	BSS Tonopen	BSS Schiotz
**2260**	16	17	12	12	15	16	16	17	14	14	14	14
2410	19	22	12	12	20	21	20	21	13	13	11	11
2740	27	26	22	22	25	26	25	26	12	12	12	13
3093	31	30	24	24	26	26	25	26	15	16	12	13
**3198**	32	32	23	24	27	28	26	26	18	17	15	14
3090	26	26	18	20	26	26	27	28	17	17	14	14
2740	18	17	16	17	25	26	22	22	18	18	17	17
2420	18	17	16	17	21	22	20	22	18	18	19	18
**2260**	17	17	14	13	20	20	18	18	17	17	14	13

*P* ^*∗*^	0.441		0.593		0.55		0.6		0.574		0.953	

*R*/*R*2^*∗∗*^	0.979/0.958		0.982/0.964		0.987/0.974		0.977/0.954		0.979/0.958		0.962/0.925	

Differences in intraocular pressure among all study eyes, according to its particular measuring method and change in altitude. IOP: intraocular pressure. SF_6_: sulfur hexafluoride. BSS: balanced saline solution. Pneum: rabbit eye without vitrectomy and 0.35 cc of 100% pure SF_6_. *P* ^*∗*^: Wilcoxon signed rank test. *R*/*R*2^*∗∗*^: Pearson's coefficient of correlation/coefficient of determination.

**Table 3:** Regression lines and slopes per case in rabbits.

Case	Regression	IOP increase/100 m altitude rise mmHg (95% CI)	Significance (95% CI) Benchmark: 100% SF_6_
100% SF_6_	*Y* = −18.67 + 1.53*x*	1.53 (0.9 to 2.2)	
50% SF_6_	*Y* = −10.70 + 1.04*x*	1.046 (0.5 to 1.6)	NS
100% Air	*Y* = −4.11 + 0.97*x*	0.9707 (0.7 to 1.2)	NS
25% SF_6_	*Y* = −3.35 + 0.97*x*	0.9716 (0.6 to 1.4)	NS
Pneum	*Y* = 11.78 + 0.14*x*	0.1485 (−0.4 to 0.7)	Sig
BSS	*Y* = 16.06 − 0.06*x*	0.0685 (−0.7 to 0.5)	Sig

Linear regression in rabbit eyes. IOP: intraocular pressure. SF_6_: sulfur hexafluoride. BSS: balanced saline solution. Pneum: rabbit eye without vitrectomy and 100% SF_6_. NS: not significant. Sig: significant.

**Table 4:** Regression lines and slopes per case and per scleral rigidity coefficient (estimates for human eyes).

Case	Regression	IOP increase/100 m altitude rise mmHg (95% CI)	Significance (95% CI) Benchmark: 100% SF_6_
Friedenwald *K* = 0.0215			
100% SF_6_	*Y* = −29.25 + 2.1*x*	2.1 (1.7 to 2.5)	
50% SF_6_	*Y* = −28.63 + 1.8*x*	1.8 (0.6 to 3.0)	NS
100% Air	*Y* = −13.23 + 1.4*x*	1.4 (0.3 to 2.5)	NS
25% SF_6_	*Y* = −5.99 + 1.1*x*	1.1 (0.2 to 2.0)	NS
Pneum	*Y* = 3.14 + 0.4*x*	0.4 (−0.4 to 1.3)	Sig
BSS	*Y* = 9.36 + 0.1*x*	0.1 (−0.6 to 0.9)	Sig

Dastiridou mean *K* = 0.0224			
100% SF_6_	*Y* = −32.16 + 2.2*x*	2.2 (1.8 to 2.6)	
50% SF_6_	*Y* = −31.16 + 1.9*x*	1.9 (0.7 to 3.1)	NS
100% Air	*Y* = −14.97 + 1.5*x*	1.5 (0.3 to 2.6)	NS
25% SF_6_	*Y* = −7.27 + 1.2*x*	1.2 (0.2 to 2.1)	NS
Pneum	*Y* = 2.64 + 0.4*x*	0.4 (−0.5 to 1.3)	Sig
BSS	*Y* = 9.19 + 0.1*x*	0.1 (−0.6 to 0.9)	Sig

Pallikaris *K* = 0.0126			
100% SF_6_	*Y* = −5.47 + 1.0*x*	1.0 (0.7 to 1.3)	
50% SF_6_	*Y* = −7.79 + 0.9*x*	0.9 (0.3 to 1.5)	NS
100% Air	*Y* = 1.28 + 0.7*x*	0.7 (0.1 to 1.3)	NS
25% SF_6_	*Y* = 4.99 + 0.6*x*	0.6 (0.1 to 1.1)	NS
Pneum	*Y* = 7.87 + 0.2*x*	0.2 (−0.3 to 0.7)	Sig
BSS	*Y* = 11.16 + 0.1*x*	0.1 (−0.4 to 0.5)	Sig

Dastiridou max *K* = 0.0343			
100% SF_6_	*Y* = −81.93 + 4.4*x*	4.4 (4.0 to 4.8)	
50% SF_6_	*Y* = −74.11 + 3.8*x*	3.8 (1.4 to 6.1)	NS
100% Air	*Y* = −43.99 + 2.8*x*	2.8 (0.9 to 4.8)	NS
25% SF_6_	*Y* = −27.51 + 2.1*x*	2.1 (0.5 to 3.7)	Sig
Pneum	*Y* = −4.25 + 0.7*x*	0.7 (−0.7 to 2.1)	Sig
BSS	*Y* = 7.1 + 0.2*x*	0.2 (−0.9 to 1.3)	Sig

Linear regression estimation for human eyes. IOP: intraocular pressure. SF_6_: sulfur hexafluoride. BSS: balanced saline solution. Pneum: rabbit eye without vitrectomy and 100% SF_6_. NS: not significant. Sig: significant.

## References

[1] Han D. P., Lewis H., Lambrou F. H., Mieler W. F., Hartz A. (1989). Mechanisms of intraocular pressure elevation after pars plana vitrectomy. *Ophthalmology*.

[2] Thompson J. T. (1989). Kinetics of intraocular gases. Disappearance of air, sulfur hexafluoride, and perfluoropropane after pars plana vitrectomy. *Archives of Ophthalmology*.

[3] Chen P. P., Thompson J. T. (1997). Risk factors for elevated intraocular pressure after the use of intraocular gases in vitreoretinal surgery. *Ophthalmic Surgery and Lasers*.

[4] Ghartey K. N., Tolentino F. I., Freeman H. M., McMeel J. W., Schepens C. L., Aiello L. M. (1980). Closed vitreous surgery. XVII. Results and complications of pars plana vitrectomy. *Archives of Ophthalmology*.

[5] Hutter J. C., Luu H. M. D., Schroeder L. W. (2002). A biological model of tamponade gases following pneumatic retinopexy. *Current Eye Research*.

[6] Abrams G. W., Swanson D. E., Sabates W. I., Goldman A. I. (1982). The results of sulfur hexafluoride gas in vitreous surgery. *American Journal of Ophthalmology*.

[7] Sabates W. I., Abrams G. W., Swanson D. E., Norton E. W. D. (1981). The use of intraocular gases. The results of sulfur hexafluoride gas in retinal detachment surgery. *Ophthalmology*.

[8] Lincoff H., Kreissig I., Coleman D. J., Chang S. (1983). Use of an intraocular gas tamponade to find retinal breaks. *American Journal of Ophthalmology*.

[9] Ruby A. J., Grand M. G., Williams D., Thomas M. A. (1999). Intraoperative acetazolamide in the prevention of intraocular pressure rise after pars plana vitrectomy with fluid-gas exchange. *Retina*.

[10] Mittra R. A., Pollack J. S., Dev S. (2000). The use of topical aqueous suppressants in the prevention of postoperative intraocular pressure elevation after pars plana vitrectomy with long-acting gas tamponade. *Ophthalmology*.

[11] Benz M. S., Escalona-Benz E. M., Murray T. G. (2004). Immediate postoperative use of a topical agent to prevent intraocular pressure elevation after pars plana vitrectomy with gas tamponade. *Archives of Ophthalmology*.

[12] Sciscio A., Casswell A. G. (2001). Effectiveness of apraclonidine 1% in preventing intraocular pressure rise following macular hole surgery. *British Journal of Ophthalmology*.

[13] Gedde S. J. (2002). Management of glaucoma after retinal detachment surgery. *Current Opinion in Ophthalmology*.

[14] Juzoji H., Iwasaki T., Usui M., Hasemi M., Yamakawa N. (1997). Histological study of intraocular changes in rabbits after intravitreal gas injection. *Japanese Journal of Ophthalmology*.

[15] Doi M., Ning M., Semba R., Uji Y., Refojo M. F. (2000). Histopathologic abnormalities in rabbit retina after intravitreous injection of expansive gases and air. *Retina*.

[16] Kreissig I. (1990). The expanding gas operation after a 15-year use. Animal experiment studies, subsequent developments of the method and clinical results in the treatment of ablatio retinae. *Klinische Monatsblätter für Augenheilkunde*.

[17] Gandorfer A., Kampik A. (2000). Expansion of intraocular gas due to reduced atmospheric pressure. Case report and review of the literature. *Der Ophthalmologe*.

[18] Fang I.-M., Huang J.-S. (2002). Central retinal artery occlusion caused by expansion of intraocular gas at high altitude. *American Journal of Ophthalmology*.

[19] Lincoff H. (2002). Transient amaurosis associated with intraocular gas during ascending high-speed train travel. *Retina*.

[20] Shiramizu K. M., Okada A. A., Hirakata A. (2001). Transient amaurosis associated with intraocular gas during ascending high-speed train travel. *Retina*.

[21] Lincoff H., Weinberger D., Stergiu P. (1989). Air travel with intraocular gas. II. Clinical considerations. *Archives of Ophthalmology*.

[22] Friberg T. R. (2001). IOP rise during simulated flight. *Ophthalmology*.

[23] Mills M. D., Devenyi R. G., Lam W.-C., Berger A. R., Beijer C. D., Lam S. R. (2001). An assessment of intraocular pressure rise in patients with gas-filled eyes during simulated air flight. *Ophthalmology*.

[24] Lincoff H., Weinberger D., Reppucci V., Lincoff A. (1989). Air travel with intraocular gas. I. The mechanisms for compensation. *Archives of Ophthalmology*.

[25] Barr C. C., Lai M. Y., Lean J. S. (1993). Postoperative intraocular pressure abnormalities in the Silicone Study. Silicone Study Report 4. *Ophthalmology*.

[26] Cekic O., Ohji M. (2000). Intraocular gas tamponades. *Seminars in Ophthalmology*.

[27] Verra S. E., Kroeze R., Ruggeri K. (2016). Facilitating safe and successful cross-border healthcare in the European Union. *Health Policy*.

[28] Tsuda T. (2014). Characteristics of atmospheric gravity waves observed using the MU (Middle and Upper atmosphere) radar and GPS (Global Positioning System) radio occultation. *Proceedings of the Japan Academy Series B: Physical and Biological Sciences*.

[29] Stevens K. B., Pfeiffer D. U. (2015). Sources of spatial animal and human health data: casting the net wide to deal more effectively with increasingly complex disease problems. *Spatial and Spatio-temporal Epidemiology*.

[30] McCrorie P. R. W., Fenton C., Ellaway A. (2014). Combining GPS, GIS, and accelerometry to explore the physical activity and environment relationship in children and young people—a review. *International Journal of Behavioral Nutrition and Physical Activity*.

[31] Cumming G. (2009). Inference by eye: reading the overlap of independent confidence intervals. *Statistics in Medicine*.

[32] Friedenwald J. S. (1937). Contribution to the theory and practice of tonometry. *American Journal of Ophthalmology*.

[33] Pallikaris I. G., Kymionis G. D., Ginis H. S., Kounis G. A., Tsilimbaris M. K. (2005). Ocular rigidity in living human eyes. *Investigative Ophthalmology and Visual Science*.

[34] Dastiridou A. I., Ginis H. S., de Brouwere D., Tsilimbaris M. K., Pallikaris I. G. (2009). Ocular rigidity, ocular pulse amplitude, and pulsatile ocular blood flow: the effect of intraocular pressure. *Investigative Ophthalmology and Visual Science*.

[35] Fraser S., Steel D. (2010). Retinal detachment. *BMJ Clinical Evidence*.

[36] Drance S. M. (1960). The coefficient of scleral rigidity in normal and glaucomatous eyes. *Archives of Ophthalmology*.

[37] Simone J. N., Whitacre M. M. (1990). The effect of intraocular gas and fluid volumes on intraocular pressure. *Ophthalmology*.

[38] Friedenwald J. S. (1947). Some problems in the calibration of tonometers. *Transactions of the American Ophthalmological Society*.

[39] Patel H., Gilmartin B., Cubbidge R. P., Logan N. S. (2011). In vivo measurement of regional variation in anterior scleral resistance to Schiotz indentation. *Ophthalmic and Physiological Optics*.

[40] Sergienko N. M., Shargorogska I. (2012). The scleral rigidity of eyes with different refractions. *Graefe's Archive for Clinical and Experimental Ophthalmology*.

